# 

**DOI:** 10.1192/bjb.2025.13

**Published:** 2025-06

**Authors:** Chrissy Jayarajah

**Affiliations:** Kensington and Chelsea Perinatal Mental Health Service, Central and North West London (CNWL) NHS Foundation Trust, London, UK. Email: c.jayarajah@nhs.net

**Figure:**
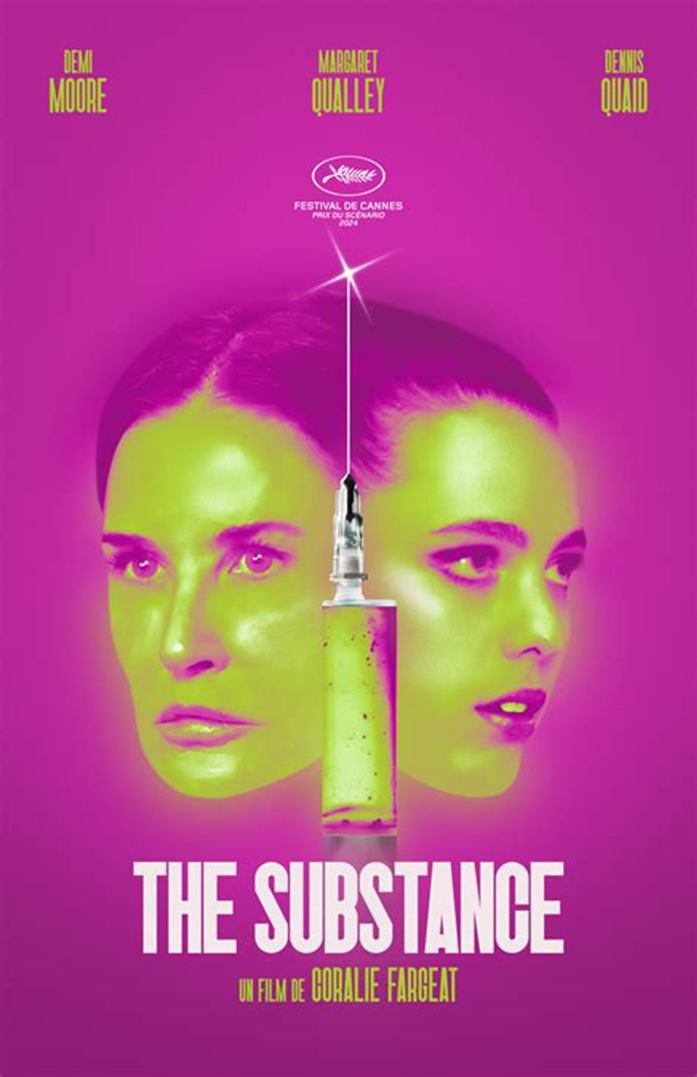

*The Substance* is a feminist body horror film set in Hollywood, where sunny streets lined with palm trees and star plaques mask a darker underbelly. The story follows Elisabeth Sparkle (Demi Moore), a fitness instructor fired from her daytime aerobics show on her 50th birthday, replaced by a ‘younger, hotter version’. Drowning her sorrows in martinis, she discovers an ad for the Substance, a fluorescent elixir promising youth and beauty. The catch? It only lasts a week, forcing users to oscillate between their older and younger selves. Tempted by nostalgia, Elisabeth injects herself and transforms into ‘Sue’ (Margaret Qualley), her vibrant, youthful alter ego. Initially, life feels perfect. However, the days in between of forced confinement in her older body drag. As time passes, the dual identities take a dark turn, with a shocking and repulsive ending, paying homage to Cronenberg and the horror movies of the 1980s.

French director Coralie Fargeat curates a visually stunning narrative with sharp cinematography and minimal dialogue, blending the artistic flair of the morbid storytelling of Wes Anderson, Stanley Kubrick and Ottessa Moshfegh. Beneath its visceral thrills, the film delivers poignant social commentary on ageing, body image and perfectionism. Elisabeth's transformation highlights a culture obsessed with beauty and youth, where older women are rendered invisible and irrelevant. Men play a peripheral yet significant role, reinforcing societal misogyny through dismissive remarks (‘pretty girls should smile’) and objectifying stares.

The film's psychoanalytical depth recalls R.D. Laing's *The Divided Self*, as we witness a woman at war within herself descend to a place of madness internally while maintaining a joyful mask externally. As Elisabeth ages, she finds compassion for her younger self, whereas in contrast, her younger self is all about having a good time and, in the spirit of those in their early 20s, feels immortal.

The body horror (also called biological horror) film subgenre plays on the boundaries of the limitations of human existence and biological function. What makes this film even more horrifying is that the story is told very much in the present day. As we live in an age preoccupied with filters and fillers (the aesthetics industry is estimated to be worth $45.5 billion worldwide), the premise of the Substance does not feel ‘science fiction’ but disturbingly factual. *The Substance* also touches on ingrained gender ideologies, reminiscent of the Wicked Witch envying Snow White's beauty. A haunting ‘magic mirror’ parallel underscores the relentless self-scrutiny women face, magnifying every wrinkle and flaw. The film's haemorrhagic climax may repel the squeamish. However, those not deterred by flesh and blood will devour this film. It's a chilling, timely masterpiece that challenges societal norms, offering a feminist horror that could shift cultural perspectives and redefine the genre.

